# Achieving High Strength and Good Ductility in As-Extruded Mg–Gd–Y–Zn Alloys by Ce Micro-Alloying

**DOI:** 10.3390/ma11010102

**Published:** 2018-01-10

**Authors:** Zhengyuan Gao, Linsheng Hu, Jinfeng Li, Zhiguo An, Jun Li, Qiuyan Huang

**Affiliations:** 1 School of Mechatronics and Automotive Engineering, Chongqing Jiaotong University, Chongqing 400074, China; linshenghucqjtu@163.com (L.H.); ljfteemo@163.com (J.L.); anzhiguo@cqjtu.edu.cn (Z.A.); cqleejun@cqjtu.edu.cn (J.L.); 2 Institute of Metal Research, Chinese Academy of Sciences, Shenyang 110016, China

**Keywords:** magnesium alloys, alloying, second phases, dynamic recrystallization, mechanical properties

## Abstract

In this study, the effect of Ce additions on microstructure evolution of Mg–7Gd–3.5Y–0.3Zn (wt %) alloys during the casting, homogenization, aging and extrusion processing are investigated, and novel mechanical properties are also obtained. The results show that Ce addition promotes the formation of long period stacking ordered (LPSO) phases in the as-cast Mg–Gd–Y–Zn–Ce alloys. A high content of Ce addition would reduce the maximum solubility of Gd and Y in the Mg matrix, which leads to the higher density of Mg12Ce phases in the as-homogenized alloys. The major second phases observed in the as-extruded alloys are micron-sized bulk LPSO phases, nano-sized stripe LPSO phases, and broken Mg12Ce and Mg5RE phases. Recrystallized grain size of the as-extruded 0.2Ce, 0.5Ce and 1.0Ce alloys can be refined to ~4.3 μm, ~1.0 μm and ~8.4 μm, respectively, which is caused by the synthesized effect of both micron phases and nano phases. The strength and ductility of as-extruded samples firstly increase and then decrease with increasing Ce content. As-extruded 0.5Ce alloy exhibits optimal mechanical properties, with ultimate strength of 365 MPa and ductility of ~15% simultaneously.

## 1. Introduction

In recent years, the demand for environment-friendly structural materials with light weight and novel mechanical properties has become urgent owing to strict emission limitations in the international community and the rapid depletion of fossil fuels [1,2]. Magnesium alloy, as the lightest metallic structural material, possesses some obvious advantages such as low density, high specific strength, notable shock absorption and noise reduction, excellent electromagnetic shielding performance, good machine ability, and recyclability [3,4]. In this sense, Mg alloy has become a promising candidate for use in various fields such as aircraft, automotives, 3D products and so on [5,6,7]. However, the industrial application of Mg alloy is still limited due to its low absolute strength and poor ductility [8]. Therefore, a popular research topic has been about the development of a new type of Mg alloy with high strength and excellent ductility, simultaneously.

The addition of rare earth (RE) elements plays an important role in purifying molten alloys and refining grains in Mg alloys. Moreover, it can induce formations of the high-density long period stacking ordered structure (LPSO) phases. Therefore, the properties of Mg alloy such as yield strength, ultimate strength, elongation and corrosion resistances, can be improved significantly with the addition of RE elements [9,10,11,12,13]. At present, Mg alloys containing the LPSO phases mainly involve ternary Mg–Zn–X alloys (X = Y, Gd, Dy, Er, Ho, Tm, Tb), as well as the quaternary Mg–Gd–Y–Zn alloys [14,15]. It is believed that the high density of the micron-sized LPSO phases induced by multi-alloying can improve the strength of the Mg alloys via the short-fiber strengthening mechanism, while the nano-sized LPSO phases in the Mg matrix can contribute to the strength and plasticity of the alloy simultaneously based on the dislocation mechanism [16,17,18,19,20]. On the other hand, previous studies have shown that the volume fraction of the micron-sized and nano-sized LPSO phases increased when the other types of the micro-alloying elements are added in the Mg–Gd–Y–Zn alloy, and the strength and ductility can be further improved. For example, Wang et al. confirmed that the LPSO phases in the as-cast Mg–2.5Zn–2.5Y–1Mn (at %) alloys were increased by Ca addition [21]. Recently, Zhang et al. showed that a small amount of Zr addition could induce a new nano-sized phase in the Mg–Gd–Zn alloy, and also change the morphology and distribution of LPSO phases, while little effect of Zr addition on the quantity of the LPSO phases can be detected [21]. In particular, Ce is an alloying element that can effectively refine the Mg matrix and also greatly reduce the stacking fault energy of the α-Mg matrix [22]. In this sense, Ce may have a significant effect on formation of the LPSO phases in the RE-containing Mg alloys. However, there are few reports on the effect of Ce alloying on the microstructure and mechanical properties of Mg–Gd–Y–Zn alloy. Therefore, in this paper, the second phase in as-cast, as-homogenized, as-aged and as-extruded Mg–Gd–Y–Zn–Ce alloys is systematically investigated, and the grain size, textures and yield strength of the alloys are also characterized, in order to provide theoretical support for the development of new high-strength and high-ductility Mg wrought alloys.

## 2. Experiment

The as-cast Mg–7Gd–3.5Y–0.3Zn–xCe (x = 0.2, 0.5, 1.0) billets were prepared by vacuum induction melting, and this was named the 0.2Ce alloy, for example, with 0.2 wt % Ce addition. The raw materials were pure magnesium (99.95 wt %), pure zinc (99.99 wt %), pure cerium (99.97 wt %), magnesium–yttrium master alloy (Mg–20 wt % Y) and Mg–Gd master alloy (Mg–20 wt % Gd). During the melting process, argon gas was used to avoid oxidation in the furnace. The molten alloys were poured into a preheated iron mold (350 °C) when the raw materials had been melted and fully stirred. Chemical analysis was performed to determine the actual compositions, and the results are listed in Table 1.

Homogenization treatments were conducted on the as-cast ingots at the temperature of 540 °C for 24 h. As-aged samples were prepared by a subsequent aging treatment at 400 °C for 1 h. After pre-heating at 400 °C for 20 min, the as-aged samples were indirectly extruded into the bars with a diameter of 10 mm under an extrusion ratio of 18:1, extrusion temperature of 400 °C and a ram speed of 0.5 mm/s. Fiel-emission scanning-electron microscopy (SEM) and an energy-dispersive spectrometer (EDS, JEOL JEM-2100F, JEOL Ltd., Osaka, Japan) were applied to reveal the microstructure of as-cast, as-homogenized, as-aged and as-extruded samples, and also the compositions of the second phases. The microstructures of the as-extruded samples were also characterized by optical microscopy (OM, ZEISS, Heidenheim, Germany), and the macro-texture of the as-extruded samples were determined by X-ray diffraction (XRD, Philips PW3040/60 X’ Pert PRO, Royal Dutch Philips Electronics Ltd., Amsterdam, The Netherlands). Finally, the mechanical properties of the samples (length of 25 mm, diameter of 5 mm) were tested under tension directions, with the initial strain rate of 10–3/s (Shimazu AG-X Plus, SHIMAZU, Kyōto-fu, Japan). The equilibrium phase diagrams of the Mg alloys were also calculated using the Panda software (Compu Therm LLC, Madison, WI, USA).

## 3. Results

### 3.1. Microstructures of the As-Cast Mg–Gd–Y–Zn–Ce Alloys

**Figure 1:**
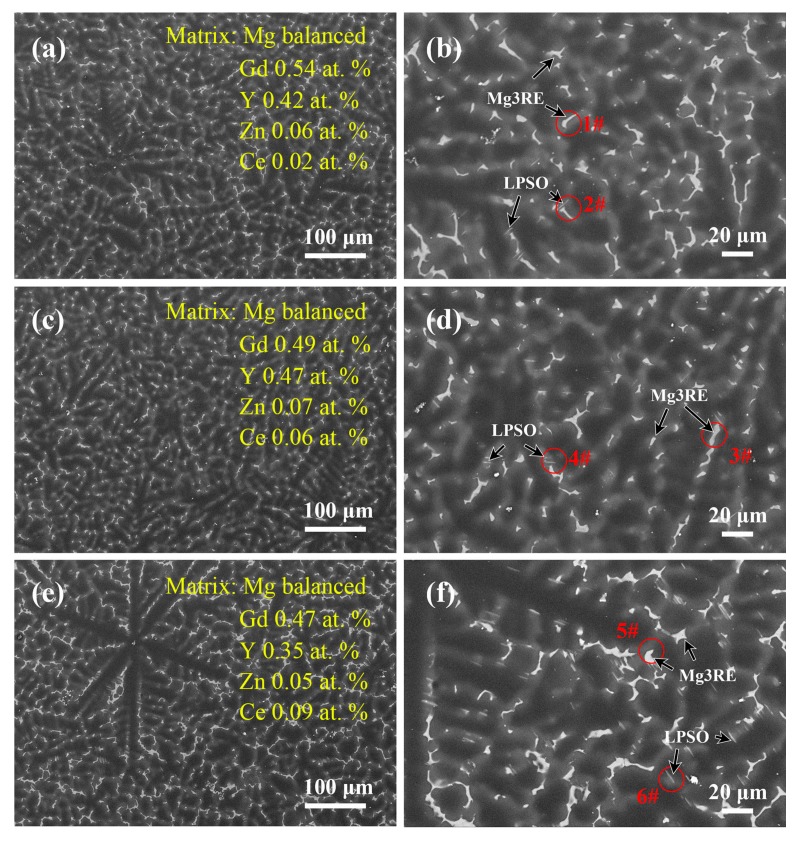
Scanning-electron microscopy (SEM) images of the as-cast Mg–7Gd–3.5Y–0.3Zn–xCe (x = 0.2, 0.5, 1.0) alloys: (**a**,**b**) 0.2Ce alloy; (**c**,**d**) 0.5Ce alloy; (**e**,**f**) 1.0Ce alloy.

The SEM images of the as-cast Mg–7Gd–3.5Y–0.3Zn–xCe (x = 0.2, 0.5, 1.0, wt %) alloys are shown in Figure 1. The as-cast 0.2Ce, 0.5Ce and 1.0Ce alloys exhibit a typical non-equilibrium solidification microstructure, containing the dendrite and dendritic segregation zones. By increasing Ce additions, the number density of irregular-shaped phases and stripe-phases increases and the size also grows gradually. As listed in Table 2, the EDS results indicate that the composition of irregular 1# second phase in the 0.2Ce as-cast alloy is Mg89.38Gd5.36Y3.5Zn1.12Ce0.63, and the 2# stripe second phase contains 2.98 at % RE (Gd, Y, Ce) and 2.54 at % Zn, which are indicated by circles in Figure 1b. Based on the morphologies and atomic ratios (RE/Zn) of the compounds, the corresponding 1# and 2# particles can be identified to be the Ce-enriched Mg_5_RE phase and the LPSO phase (RE/Zn ≈ 1), respectively. These results are in agreement with previous reports on the second phases formed in the as-cast Mg–Gd–Y–Zn based alloys [12,13]. Moreover, EDS analysis of the α-Mg matrix shows higher content of dissolved Ce solute with increasing Ce additions.

The size and distribution of the Ce-enriched Mg_5_RE phase and LPSO phase in the as-cast 0.5Ce alloy is similar to that in the as-cast 0.2Ce alloy, while the fraction of the LPSO phase and the dissolved content of Ce atoms increase slightly. In the as-cast 1Ce alloy, on the other hand, the size of the Ce-enriched Mg_5_RE phase increases significantly, and the quantity of the LPSO phase also becomes obviously higher.

### 3.2. Microstructures of the As-Homogenized and As-Aged Mg–Gd–Y–Zn–Ce Alloys

**Figure 2:**
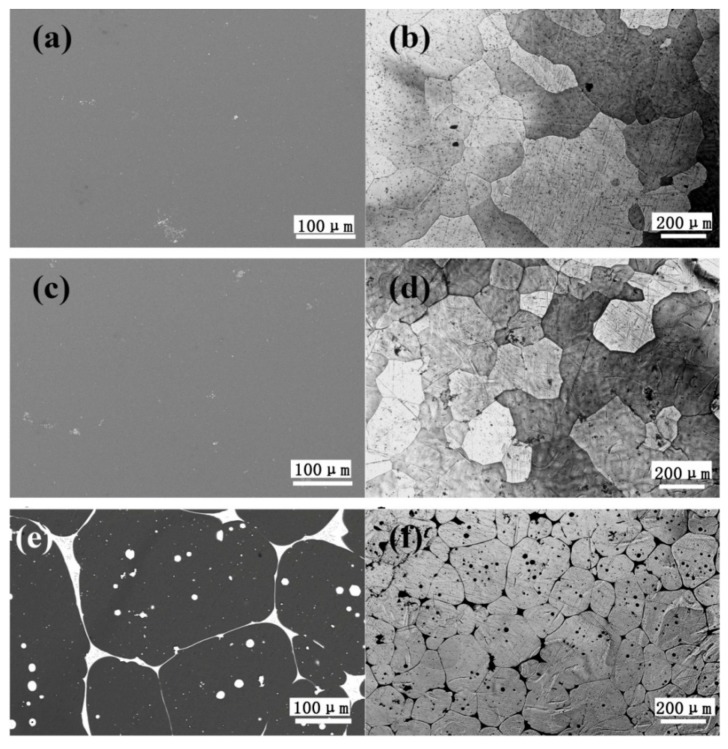
(**a**,**c**,**e**) SEM images and (**b**,**d**,**f**) optical images of the as-homogenized Mg–7Gd–3.5Y–0.3Zn–xCe (x = 0.2, 0.5, 1.0) alloys: (**a**,**b**) 0.2Ce alloy; (**c**,**d**) 0.5Ce alloy; (**e**,**f**) 1.0Ce alloy.

**Figure 3:**
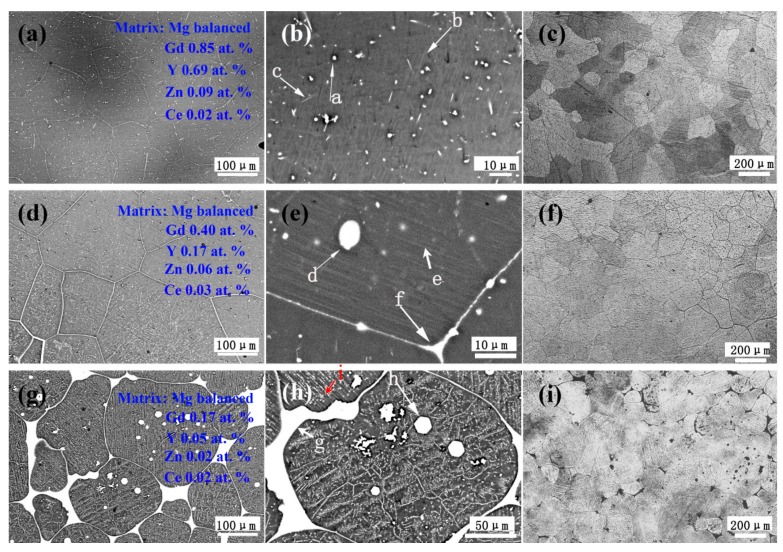
(**a**,**b**,**d**,**e**,**g**,**h**) SEM images and (**c**,**f**,**i**) optical images of the as-aged Mg–7Gd–3.5Y–0.3Zn–xCe (x = 0.2, 0.5, 1.0) alloys: (**a**,**b**,**c**) 0.2Ce alloy; (**d**,**e**,**f**) 0.5Ce alloy; (**g**,**h**,**i**) 1.0Ce alloy; (**a**,**d**,**g**) lower-magnification images; and (**b**,**e**,**h**) higher-magnification images.

Figure 2 shows the SEM and optical microscopy images of the as-homogenized 0.2Ce, 0.5Ce and 1.0Ce alloys. The second phases and the segregation zones in the as-cast 0.2Ce and 0.5Ce alloys have been almost dissolved by the high temperature solid solution treatment. In contrast, a large number of reticular and spherical second phases still exist along the grain boundaries and within the grain interior of the as-homogenized 1.0Ce alloy. According to the EDS results, the second phase is the Mg_12_Ce phase enriched with RE elements. At the same time, the grain size of as-homogenized 0.2Ce, 0.5Ce and 1.0Ce samples decreases gradually with the increasing addition of Ce, i.e., ~370 μm, ~260 μm and ~160 μm, respectively. As shown in Figure 3, after the subsequent aging treatment of the as-homogenized samples, numerous precipitations can be detected in the 0.2Ce, 0.5Ce and 1.0Ce alloys, including the reticular, spherical and stripe phases. The EDS results (Table 3) show that the spherical second phase “a” in Figure 3b contains ~3.4 at % Gd, ~9.21 at % Y and a small amount of Zn and Ce; the stripe second phase “b” contains ~2.73 at % Gd/Y, ~2.28 at % Zn and a small amount of Ce (RE/Zn ≈ 1); the reticular second phase “c” contains ~2.7 at % Gd/Y, ~1.5 at % Ce and a small amount of Zn. Therefore, the second phase “a” and “c” that precipitates in the as-aged 0.2Ce alloy can be clarified to be the Ce-enriched Mg_5_RE phase, while the second phase “b” is determined as the LPSO phase. The length and volume fraction of nano-LPSO phase in as-aged 0.2Ce alloy is estimated to be ~7 μm and ~2%, respectively.

With increasing content of Ce, both the size and number density of the second phases precipitated from the 0.5Ce alloy and the 1.0Ce alloy increase significantly (Figure 3e,h). The EDS results (Table 3) indicate that the spherical phase “d” in 0.5Ce alloy should be the Ce-enriched Mg_5_RE phases due to the high RE content, and the stripe second phases “e” are also the LPSO phases, while the reticular phase “f” should be the Mg_12_Ce phase due to the high Ce content. In other words, the addition of Ce would induce formation of the new phase of Mg_12_Ce in present Mg–Gd–Y–Zn alloys. The quantity of the Mg_12_Ce phases and the LPSO phases in 1.0Ce alloy increases significantly, as shown in Figure 3h. Volume fractions of the nano-LPSO phases in as-aged 0.5Ce and 1.0Ce alloys have increased to ~4% and ~9%, respectively. Moreover, the EDS analysis shows that the remaining Gd and Y elements dissolved in the α-Mg matrix obviously decrease with increasing Ce additions. 

### 3.3. Microstructures and Mechanical Properties of the As-Extruded Mg–Gd–Y–Zn–Ce Alloys

**Figure 4:**
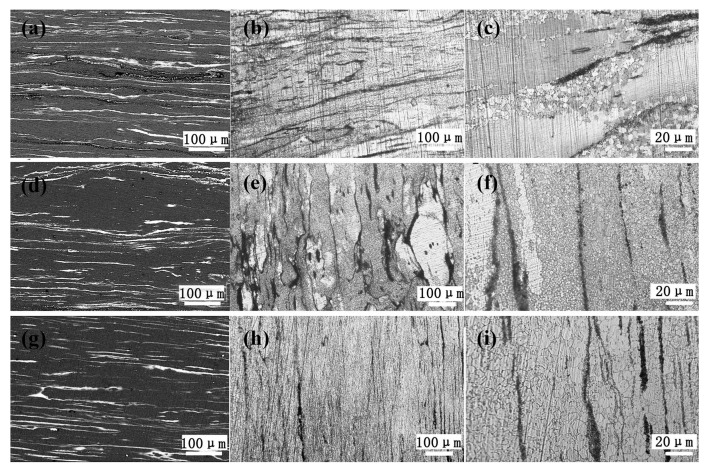
(**a**,**d**,**g**) SEM images and (**b**,**c**,**e**,**f**,**h**,**i**) optical images of the as-extruded Mg–7Gd–3.5Y–0.3Zn–xCe (x = 0.2, 0.5, 1.0) alloys: (**a**,**b**,**c**) 0.2Ce alloy; (**d**,**e**,**f**) 0.5Ce alloy; (**g**,**h**,**i**) 1.0Ce alloy; (**b**,**e**,**h**) lower-magnification images; and (**c**,**f**,**i**) higher-magnification images.

**Figure 5:**
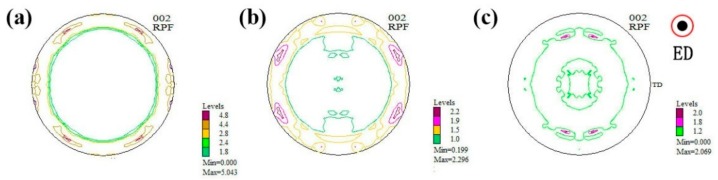
The (0001) pole figures of the as-extruded Mg–7Gd–3.5Y–0.3Zn–xCe (x = 0.2, 0.5, 1.0) alloys: (**a**) 0.2Ce alloy; (**b**) 0.5Ce alloy; (**c**) 1.0Ce alloy.

Figure 4 shows the microstructures of as-extruded 0.2Ce, 0.5Ce and 1.0Ce alloys. As compared with the as-homogenized samples, a large number of micron-size second phases precipitate in the as-extruded alloys and distribute along the extrusion direction after the hot extrusion process (Figure 4a,d,g). Optical images show that dynamic recrystallization (DRX) occurs in the as-extruded 0.2Ce, 0.5Ce and 1.0Ce alloys, and volume fraction of the recrystallized grains is ~65%, ~75% and ~97%, respectively (Figure 4b,e,h); that is, the proportion of recrystallization increases with the increasing addition of Ce. At the same time, the coarsely as-deformed regions are compressed to be the streamline, which is aligned with the extrusion direction. The DRXed grain sizes of the 0.2Ce, 0.5Ce and 1.0Ce alloys are estimated to be ~4.3 μm, ~1.0 μm and ~8.4 μm, respectively, according to the higher magnification optical images (Figure 4c,f,i). It can be noted that grain sizes of the non-DRXed grains are not considered due to the severely heterogeneous microstructure. On the other hand, there usually exists a relationship between the macro-texture and the dynamic recrystallization behavior of the as-extruded Mg alloys. In general, DRXed grains of the RE-containing Mg alloys display a “rare earth” texture, in which the c-axis deviates from the radial direction of the extrusion rod to the extrusion direction [15,23]. Therefore, the more dynamic the recrystallization that takes place in rare earth Mg alloys, the more obvious the “rare earth” texture that would be generated. As shown in Figure 5, the macro-texture of the as-extruded 0.2Ce, 0.5Ce and 1.0Ce alloys agrees well with the statement above. Due to the high proportion of recrystallized grains and the concurrent existence of non-recrystallized grains, the “rare earth” texture and also the fiber texture co-exist in the as-extruded 0.2Ce alloy (Figure 5a). The fiber texture shows the typical texture of the non-recrystallized grains in which the c-axis is aligned with the radial direction of the bar [23]. 

With increasing addition of Ce, a higher degree of recrystallization takes place, and the c-axis tilts to the extrusion direction gradually, as shown in Figure 5b,c. The fiber texture component in the 1.0Ce alloy has basically disappeared, and only the “rare earth” texture remains, which is consistent with the complete recrystallization as detected in Figure 4. 

**Figure 6:**
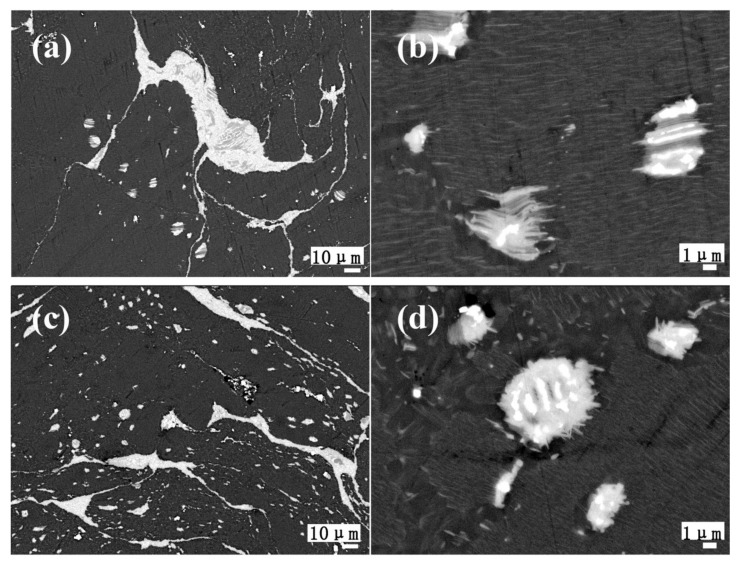

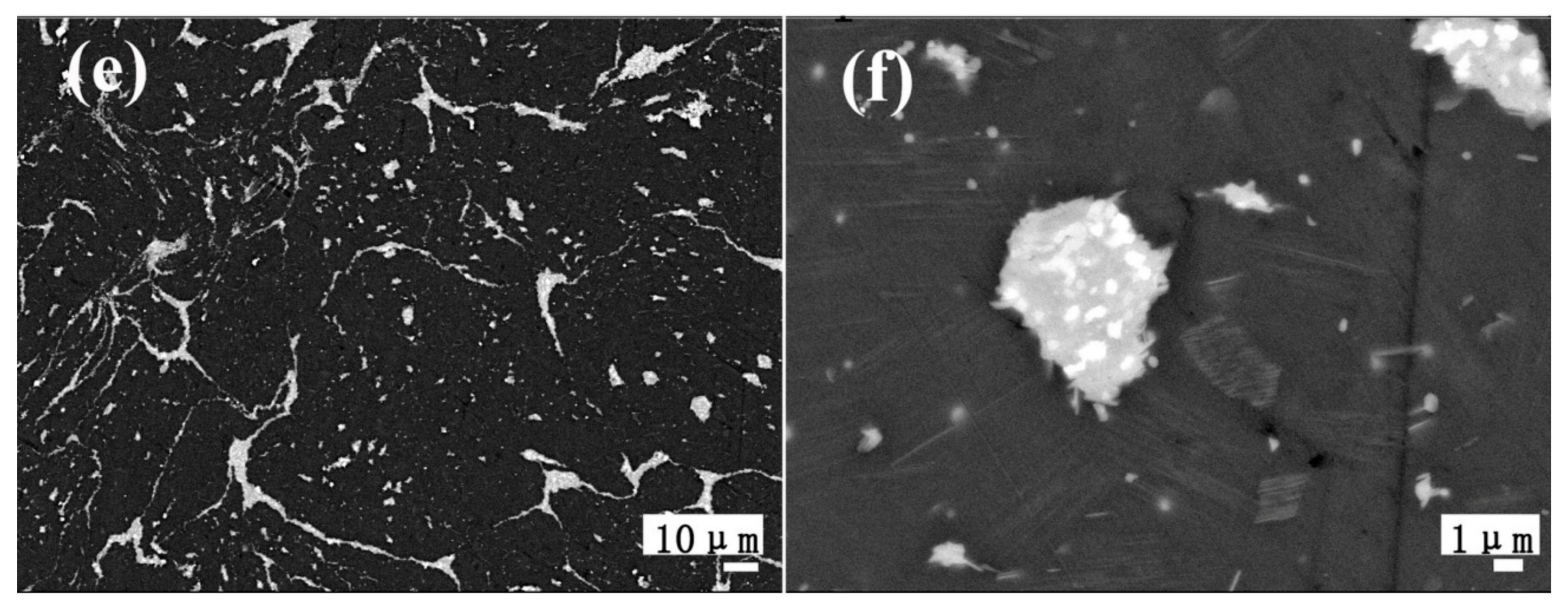
SEM images of the as-extruded Mg–7Gd–3.5Y–0.3Zn–xCe (x = 0.2, 0.5, 1.0) alloys: (**a**,**b**) 0.2Ce alloy; (**c**,**d**) 0.5Ce alloy; (**e**,**f**) 1.0Ce alloy.

As mentioned above, the morphology of the second phases varies with different amounts of Ce addition in the as-homogenized samples. Accordingly, different precipitation behaviors of the second phases are also generated in the as-extruded alloys, as shown in SEM images of as-extruded 0.2Ce, 0.5Ce and 1.0Ce alloys (Figure 6). Cross-sectional scanning results show that a large number of the non-fragmented reticular and spherical micron-size second phases are uniformly distributed in the matrix (Figure 6a,c,e), which coincides with the results of longitudinal-section scanning in Figure 4. Based on the characteristic “twisted” morphology of the second phases, these bulk micron-size phases can be identified as LPSO phases [12,13]. In order to clarify the effect of the second phase on dynamic recrystallization, higher-magnification images are also displayed and a large number of nano-sized stripe second phase can be found to disperse in the grain interior of the Mg alloys (Figure 6b,d,f). The nano-phases are similar to the nano-LPSO phases formed in the as-aged samples. Thus, it can be concluded that during hot extrusion deformation, both bulk LPSO phases and stripe nano-LPSO phases precipitated from the matrices of the 0.2Ce, 0.5Ce and 1.0Ce alloys. However, both the size and distribution of the bulk and nano-LPSO phases in the three groups of alloys are different from each other. The density and fraction of the stripe LPSO phases are high in the 0.2Ce and 0.5Ce alloys, while the stripe LPSO phase exists in the form of clusters with lower inter-distances in the 1.0Ce alloy and the volume fraction obviously decreases (Table 4). Moreover, the micron-sized Mg_12_Ce phases are broken into spherical particles in the 0.5Ce and 1.0Ce alloys, and co-exist with the Mg_5_RE phases. With increasing Ce addition, the corresponding volume fraction of the spherical phases also increases.

**Figure 7:**
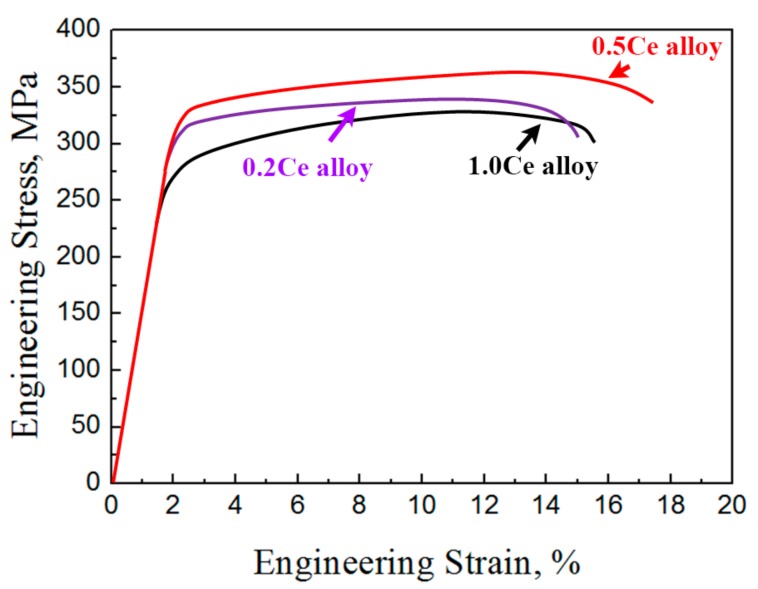
Engineering stress-strain curves of the as-extruded Mg–7Gd–3.5Y–0.3Zn–xCe (x = 0.2, 0.5, 1.0) alloys.

The mechanical properties of the as-extruded samples under both tensile and compressive conditions are shown in Figure 7 and Table 5. Under tensile deformation, the yield strength (YS) and ultimate strength (UTS) of as-extruded 0.2Ce alloy are 286.4 MPa and 340.7 MPa, respectively. With the addition of 0.5 wt % Ce, the YS and UTS increase to 302.2 MPa and 365.3 MPa. For as-extruded 1.0Ce alloy, the YS and UTS decrease to 263.5MPa and 327.3MPa, respectively. In addition, the elongation to fracture of the as-extruded alloy increases from ~13% in 0.2Ce alloy to ~15% in 0.5Ce alloy, and then decreases to ~12% in the as-extruded 1.0Ce alloy. The strength and ductility of the as-extruded alloys exhibit a trend of initial increase and then decrease with increasing Ce addition. The YS, UTS and elongation of the as-extruded 0.5Ce alloy are the highest among the three samples. In other words, the addition of a small amount of Ce is beneficial for improving mechanical properties of as-extruded Mg–7Gd–3.5Y–0.3Zn alloys, and the optimal Ce content is about 0.5 wt %.

## 4. Discussion

**Figure 8:**
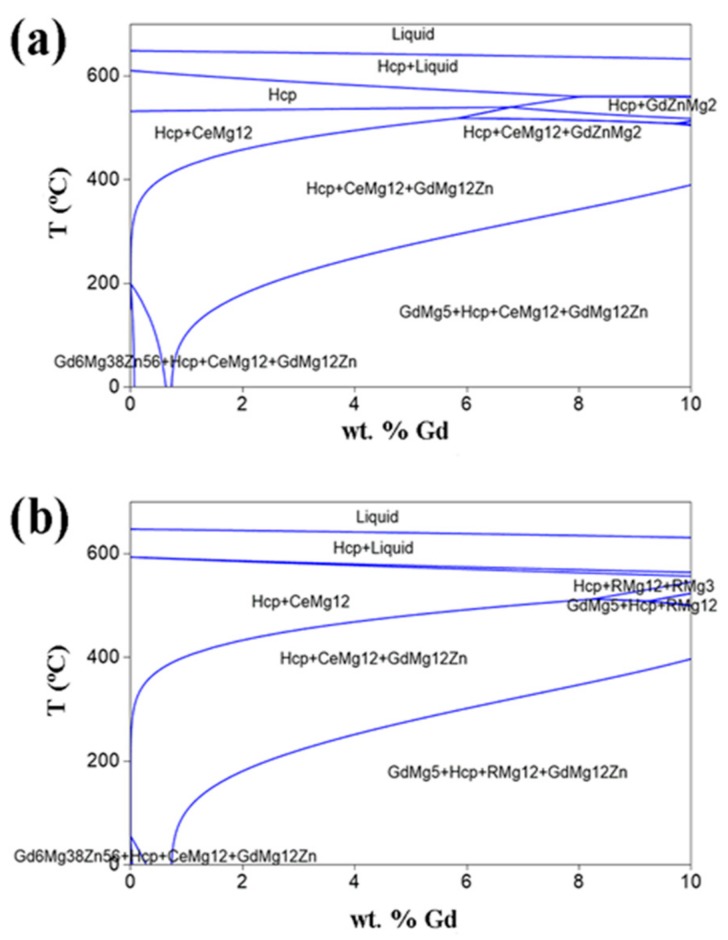
The polythermic sections for the equilibrium phase diagrams of the (**a**) Mg–0.3Zn–0.2Ce–xGd (x = 0–10 wt %) and (**b**) Mg–0.3Zn–1.0Ce–xGd (x = 0–10 wt %) alloys.

As-cast samples exhibit the typical non-equilibrium microstructure, and the main second phases are Ce-enriched Mg_5_RE and LPSO phases. With the addition of Ce, not only does the size of Mg_5_RE phases increase, but also the fraction of LPSO phases increases gradually, which means that Ce addition can promote the formation of LPSO phases in the as-cast Mg–Gd–Y–Zn alloys. This may be attributed to the mechanism whereby Ce reduces the stacking fault energy of the Mg matrix effectively [22]. After solid solution treatment, the eutectic microstructures of as-cast 0.2Ce and 0.5Ce alloys have almost disappeared, while large numbers of RE-enriched Mg_12_Ce phases remain in the 1.0Ce alloy. Consequently, the maximum solid solubility of Gd and Y in the Mg matrix would be reduced because of the addition of a large amount of Ce, as confirmed by the EDS analysis on the α-Mg matrix. In fact, Figure 8 displays the polythermic sections for the equilibrium phase diagrams of the Mg–0.3Zn–0.2Ce–xGd (x = 0–10 wt %) and Mg–0.3Zn–1.0Ce–xGd (x = 0–10 wt %) alloys. It can clearly be seen that Ce addition would decrease the maximum solubility of the Gd elements, and also induce formation of the new Mg_12_Ce phase.

During the subsequent extrusion process, the bulk micron-sized LPSO phases dynamically precipitate at the grain interiors and boundaries of the as-received 0.2Ce, 0.5Ce and 1.0Ce alloys, as shown in Figure 6. The micron-sized Mg_12_Ce and Mg_5_RE phases in the 0.5Ce and 1.0Ce alloys have been broken into spherical particles in the conditions of thermo-mechanical processing. Consistent with the mechanism of particle-stimulated nucleation (PSN), these micron-sized particles will promote the dynamic recrystallization of the Mg matrix [15,23]. On the other hand, a large number of nano-sized stripe LPSO phases also precipitate at the grain interiors (Table 4). The second phases with inter-spacing of ~100 nm can bring an obvious drag effect on the DRXed grain boundaries, and then growths of the DRXed grains can be inhibited [12,13]. After the extrusion deformation, the 0.2Ce alloy exhibits a bimodal grain structure composed of coarse grains and ultra-fine grains. When the content of Ce is increased to 0.5 wt %, the volume fraction of the bulk LPSO phase, the micron-sized Mg_12_Ce and the Mg_5_RE phases increase. As a result, the nucleation sites of dynamic recrystallization increase. At the same time, a high content of Ce promotes the dynamic precipitation of the higher density of stripe LPSO phases (Table 4), and enhances the dragging effect on the mobility of the grain boundaries. Consequently, a higher fraction of recrystallization occurs, and the ultrafine grains (only ~1.0 μm) are formed in the as-extruded 0.5Ce alloy. With the addition of Ce increasing to 1 wt %, more Gd, Y and other elements will combine with Ce, in the form of bulk LPSO and Mg_5_RE phases, which exist in the interior or boundaries of the grains. As a result, the driving pressure for recrystallization via the PSN mechanism increases during the extrusion process. However, the precipitation of a larger number of bulk LPSO phases leads to the decrease of the residual Gd and Y elements in the Mg matrix (Table 4), and consequently the fraction of nano-sized stripe LPSO phase is obviously reduced. The inhibition effect on growth of the recrystallized grains is weakened, which leads to the formation of larger recrystallized grains in 1.0Ce alloy (~8.5 μm).

Grain refinement is an effective approach for improving the strength and ductility of Mg alloys [24]. The recrystallized grains of the as-extruded 0.2Ce alloy can be refined to ~4.3 μm, and thus they can exhibit high strength and ductility simultaneously. At the same time, the as-extruded 0.2Ce alloy also displays the characteristics of a bimodal grain structure, in which the coarse grains will improve the strength and plasticity of the alloy via the back-stress hardening mechanism [25]. For instance, Wu et al. [25] revealed that, based on the bimodal grain structure, the yield strength of titanium alloy was increased by three times, and elongation was maintained at ~8%. On the other hand, there are a large number of reticular LPSO phases and nano-sized LPSO phases in the as-extruded alloys (Table 4), which would enhance the strength and ductility of the alloys by fiber-strengthening and dispersion-strengthening mechanisms [3,17]. Recently, Hagihara et al. [14] also confirmed the strengthening effect of fiber-shaped LPSO phases in the Mg alloy. When the content of Ce increases to 0.5 wt %, the size of recrystallized grains of the alloy is refined to ~1.0 μm, the yield strength of the alloy is improved effectively based on the mechanism of grain-refinement strengthening, and the plasticity can also be improved due to the existence of the bimodal grain structure. At the same time, the density of the bulk and stripe LPSO phases increase, which can further improve the strength of the alloy. With increasing Ce addition to 1 wt %, strength and ductility will be reduced due to the growth of recrystallized grains (~8.4 μm) and the lower density of the nano-sized stripe LPSO phases (~2%, Table 4). In this sense, Ce addition can change the size and distribution of second phases in present alloys, and has an effect on the dynamic recrystallization behavior during the extrusion process to then enhance the mechanical properties of the as-extruded alloys.

## 5. Conclusions

In this work, the effects of Ce alloying on the microstructure and mechanical properties of as-cast, as-homogenized, as-aged and as-extruded Mg–7Gd–3.5Y–0.3Zn alloys have been investigated, and the following conclusions can be drawn:

(1) Ce addition promotes the formation of LPSO phases in the as-cast Mg–Gd–Y–Zn–Ce alloys. The high content of Ce addition reduces the maximum solubility of Gd and Y in the Mg matrix, and leads to a higher density of Mg_12_Ce phases in the as-homogenized alloys;
(2) the main second phases in the as-extruded alloys are bulk micron-sized LPSO phases, nano-sized stripe LPSO phases and broken Mg_12_Ce and Mg_5_RE phases. The recrystallized grain size of the as-extruded 0.2Ce, 0.5Ce and 1.0Ce alloys can be refined to ~4.3 μm, ~1.0 μm and ~8.4 μm, respectively. This can be attributed to the synthesized effect of the driving force of the micron phases and the dragging effect of the nano phases on dynamic recrystallization;
(3) with the increasing content of Ce, tension, strength and ductility of as-extruded samples would firstly increase and then decrease. The as-extruded 0.5Ce alloy exhibits optimal mechanical properties.

## Figures and Tables

**Table 1:** Compositions of the as-cast Mg–7Gd–3.5Y–0.3Zn–xCe (x = 0.2, 0.5, 1.0) alloys in weight percentage.

Samples	Gd	Y	Zn	Ce
0.2Ce alloy	7.68	3.77	0.3	0.21
0.5Ce alloy	6.77	3.42	0.3	0.47
1.0Ce alloy	7.55	3.69	0.28	1.05

**Table 2:** Energy-dispersive spectrometry (EDS) results in as-cast Mg–7Gd–3.5Y–0.3Zn–xCe (x = 0.2, 0.5, 1.0) alloys (at %).

Points	Mg	Gd	Y	Zn	Ce
1#	89.38	5.36	3.50	1.12	0.63
2#	94.47	1.11	1.86	2.54	0.01
3#	88.29	5.94	4.65	0.55	0.58
4#	93.0	1.46	1.72	3.61	0.22
5#	87.14	5.46	4.19	1.74	1.47
6#	92.01	1.89	1.82	3.90	0.38

**Table 3:** EDS results in as-aged Mg–7Gd–3.5Y–0.3Zn–xCe (x = 0.2, 0.5, 1.0) alloys (at %).

Points	Mg	Gd	Y	Zn	Ce
a	86.79	3.4	9.21	0.46	0.15
b	94.94	1.04	1.69	2.28	0.05
c	97.13	1.20	1.50	0.09	1.5
d	87.00	3.05	9.34	0.23	0.38
e	92.62	1.1	2.28	3.38	0.62
f	86.57	4.47	3.80	3.27	3.89
g	80.46	7.07	6.64	0.5	5.33
h	58.87	8.94	30.45	0.66	1.08
i	88.13	3.64	2.94	3.06	2.24

**Table 4:** Sizes of nano-long period stacking ordered structure (LPSO) and bulk LPSO, the fraction of second phases and the DRXed grain size of as-extruded Mg–7Gd–3.5Y–0.3Zn–xCe (x = 0.2, 0.5, 1.0) alloys.

As-Extruded Samples	Length of Nano-LPSO Phase	Length of Bulk LPSO Phase	Width of Bulk LPSO Phase	Fraction of Second Phases	DRXed Grain Sizes
0.2Ce alloy	~3.0 μm	~300 μm	~35 μm	4% (nano) 10% (micron)	~4.3 μm
0.5Ce alloy	~1.5 μm	~260 μm	~25 μm	5% (nano) 11% (micron)	~1.7 μm
1.0Ce alloy	~1.0 μm	~200 μm	~15 μm	2% (nano) 16% (micron)	~8.4 μm

**Table 5:** Mechanical properties of as-extruded Mg–Gd–Y–Zn–Ce alloys under tension.

Samples	Yield Strength (MPa)	Ultimate Strength (MPa)	Elongation (%)
0.2Ce alloy (tension)	286.4	340.7	13
0.5Ce alloy (tension)	302.2	365.3	15
1.0Ce alloy (tension)	263.5	327.3	13.5
